# Gastric outlet obstruction at Bugando Medical Centre in Northwestern Tanzania: a prospective review of 184 cases

**DOI:** 10.1186/1471-2482-13-41

**Published:** 2013-09-25

**Authors:** Hyasinta Jaka, Mabula D Mchembe, Peter F Rambau, Phillipo L Chalya

**Affiliations:** 1 Department of Internal Medicine, Catholic University of Health and Allied Sciences- Bugando, Mwanza, Tanzania; 2 Endoscopic unit, Bugando Medical Center, Mwanza, Tanzania; 3 Department of Surgery, Muhimbili University of Health and Allied Sciences, Dar Es Salaam, Tanzania; 4 Department of Pathology, Catholic University of Health and Allied Sciences- Bugando, Mwanza, Tanzania; 5 Department of Surgery, Catholic University of Health and Allied Sciences- Bugando, Mwanza, Tanzania

**Keywords:** Gastric outlet obstruction, Etiological spectrum, Treatment, Outcome, Tanzania

## Abstract

**Background:**

Gastric outlet obstruction poses diagnostic and therapeutic challenges to general surgeons practicing in resource-limited countries. There is a paucity of published data on this subject in our setting. This study was undertaken to highlight the etiological spectrum and treatment outcome of gastric outlet obstruction in our setting and to identify prognostic factors for morbidity and mortality.

**Methods:**

This was a descriptive prospective study which was conducted at Bugando Medical Centre between March 2009 and February 2013. All patients with a clinical diagnosis of gastric outlet obstruction were, after informed consent for the study, consecutively enrolled into the study. Statistical data analysis was done using SPSS computer software version 17.0.

**Results:**

A total of 184 patients were studied. More than two-third of patients were males. Patients with malignant gastric outlet obstruction were older than those of benign type. This difference was statistically significant (p < 0.001). Gastric cancer was the commonest malignant cause of gastric outlet obstruction where as peptic ulcer disease was the commonest benign cause. In children, the commonest cause of gastric outlet obstruction was congenital pyloric stenosis (13.0%). Non-bilious vomiting (100%) and weight loss (93.5%) were the most frequent symptoms. Eighteen (9.8%) patients were HIV positive with the median CD 4+ count of 282 cells/μl. A total of 168 (91.3%) patients underwent surgery. Of these, gastro-jejunostomy (61.9%) was the most common surgical procedure performed. The complication rate was 32.1 % mainly surgical site infections (38.2%). The median hospital stay and mortality rate were 14 days and 18.5% respectively. The presence of postoperative complication was the main predictor of hospital stay (p = 0.002), whereas the age > 60 years, co-existing medical illness, malignant cause, HIV positivity, low CD 4 count (<200 cells/μl), high ASA class and presence of surgical site infection significantly predicted mortality ( p< 0.001). The follow up of patients was generally poor as more than 60% of patients were lost to follow up.

**Conclusion:**

Gastric outlet obstruction in our setting is more prevalent in males and the cause is mostly malignant. The majority of patients present late with poor general condition. Early recognition of the diagnosis, aggressive resuscitation and early institution of surgical management is of paramount importance if morbidity and mortality associated with gastric outlet obstruction are to be avoided.

## Background

Gastric Outlet Obstruction implies complete or incomplete obstruction of the distal stomach, pylorus or proximal duodenum [1]. This may occur as an obstructing mass lesion, external compression or as a result of obstruction from acute edema, chronic scarring and fibrosis or a combination of both [1,2]. Gastric outlet obstruction is not a single entity; it is the clinical and pathophysiological consequence of any disease process that produces a mechanical impediment to gastric emptying [3].

Globally, the incidence of gastric outlet obstruction has been reported to be less than 5% in patients with peptic ulcer disease, which is the leading benign cause of the problem, whereas the incidence of gastric outlet obstruction in patients with peripancreatic malignancy, the most common malignant etiology, has been reported as 15-20% [3-5].

Gastric Outlet Obstruction may be caused by a heterogeneous group of diseases that include both benign and malignant conditions [1,6]. In children, the condition is commonly caused by pyloric stenosis which refers to a narrowing of the pylorus, the opening from the stomach into the small intestine. In adults, mechanical obstruction due to ulcers, tumors or gastric polyps are common causes of gastric outlet obstruction [7]. In the past, when peptic ulcer disease was more prevalent, benign causes were the most common; however, one review shows that only 37% of patients with gastric outlet obstruction have benign disease and the remaining patients have obstruction secondary to malignancy [3-5].

The management of gastric outlet obstruction poses diagnostic and therapeutic challenges to general surgeons practicing in resource-limited countries. Late presentation of the disease coupled with lack of modern diagnostic and therapeutic facilities are among the hallmarks of the disease in developing countries [6]. The outcome of treatment of gastric outlet obstruction may be poor especially in developing countries where advanced diagnostic and therapeutic facilities are not readily available in most centers [6,7].

Gastric outlet obstruction is usually a preterminal event in patients with advanced malignancies of the stomach, pancreas, and duodenum. Surgery is associated with a high complication rate, and relatively high morbidity and mortality rates, due to poor nutrition and general status or progressing tumor infiltration in these patients [8]. The use of self-expandable metal stents to treat gastric outlet obstructions have been demonstrated to be an effective alternative to surgical bypass with lower morbidity and mortality rates, shorter hospitalization, and a lower cost of the overall treatment [9,10]. However, these facilities are not usually available in most centers in developing countries including Tanzania,

Despite increase in the number of admissions of these patients in our setting, no clinical study has been done to analyze this problem. This study was undertaken to highlight the etiological spectrum and treatment outcome of gastric outlet obstruction in our setting and to identify prognostic factors for morbidity and mortality.

## Methods

### Study design and setting

Between March 2009 and February 2013, a descriptive prospective study involving all patients with a clinical diagnosis of gastric outlet obstruction was conducted at Bugando Medical Centre. Bugando Medical Centre is located in Mwanza city along the shore of Lake Victoria in the northwestern part of Tanzania. It is a tertiary care and teaching hospital for the Catholic University of Health and Allied Sciences-Bugando (CUHAS-Bugando) and other paramedics and has a bed capacity of 1000. Bugando Medical Centre is one of the four largest referral hospitals in the country and serves as a referral centre for tertiary specialist care for a catchment population of approximately 13 million people.

### Study population

All patients with a clinical diagnosis of gastric outlet obstruction seen at Bugando Medical Centre during the study period were consecutively included into the study.Patients with gastroparesis without any mechanical obstruction or a previously known cancer were excluded from the study. Patients who failed to consent for HIV testing were also excluded from the study. The diagnosis of gastric outlet obstruction was based on clinical presentation, an upper gastrointestinal barium study, and/or an inability during upper endoscopy to intubate the second portion of the duodenum.

Preoperatively, all the patients recruited into the study had intravenous fluids to correct fluid and electrolyte deficits; nasogastric suction; urethral catheterization and broad-spectrum antibiotic coverage. They had pre-operative anaesthetic assessment using the American Society of Anesthetists (ASA) classification [11]. Adequate hydration was indicated by an hourly urine output of 30 ml/hour.

Relevant preoperative laboratory investigations included complete blood count, hemoglobin levels, serum albumin, serum electrolytes, urea and creatinine, HIV testing (using Tanzania HIV Rapid Test Algorithm) [12] and CD 4+ count (using FACS or FACSCALIBUR from BD Biosciences USA). Imaging investigations included plain abdominal x-rays, barium studies, abdominal ultrasound and Computerized tomography scan. The diagnosis of gastric outlet obstruction was confirmed by upper gastrointestinal endoscopy and intra-operative finding.

Intra-operatively, all patients, under general anesthesia were subjected to exploratory laparotomy through midline incision. At operation, the diagnosis of gastric outlet obstruction was made by noting a cicatrized first part of duodenum or pylorus with a dilated and thick-walled stomach. The type of surgical procedure was done according to whether the cause of gastric outlet obstruction was benign or malignant. The operations were performed either by a consultant surgeon or a senior resident under the direct supervision of a consultant surgeon.

Biopsy was taken from either a mass of peripyloric lymph nodes or any gastric mass for histological examination. Intraoperative tissue biopsy was taken for histopathological studies; a portion of the tissue was fixed in 10 per cent formalin; routine processing was done as per standard operative procedures and stained with haemotoxylin and eosin.

Postoperatively patients were kept nil orally till return of bowl sounds and at that time nasogastric tubes were removed. Intravenous antibiotics were used for up to three day and continue with oral antibiotics. The postoperative outcome was monitored; patients in ASA classes IV and V were admitted into intensive care unit after surgery. Data on each patient were entered into a questionnaire prepared for the study. The study variables included socio-demographic (i.e. age and sex, level of education, occupation and area of residence), clinical presentation, HIV status, laboratory, radiological and endoscopic findings, ASA classification, operative findings and surgical procedure performed. The variables studied in the postoperative period were postoperative complications, hospital stay and mortality. Patients were followed up for a period of twelve months or till death whichever is earlier.

### Statistical analysis

The statistical analysis was performed using statistical package for social sciences (SPSS) version 17.0 for Windows (SPSS, Chicago IL, U.S.A). The median and ranges were calculated for continuous variables whereas proportions and frequency tables were used to summarize categorical variables. Continuous variables were categorized. Chi-square (χ2) test were used to test for the significance of association between the independent (predictor) and dependent (outcome) variables in the categorical variables. The level of significance was considered as P < 0.05. Multivariate logistic regression analysis was used to determine predictor variables that predict the outcome.

### Ethical consideration

Ethical approval to conduct the study was obtained from the Catholic University of Health and Allied Sciences-Bugando/Bugando Medical Center joint institutional ethic review committee before the commencement of the study. Patients were required to sign a written informed consent for the study and for HIV testing.

## Results

### Patient’s characteristics

**Figure 1:**
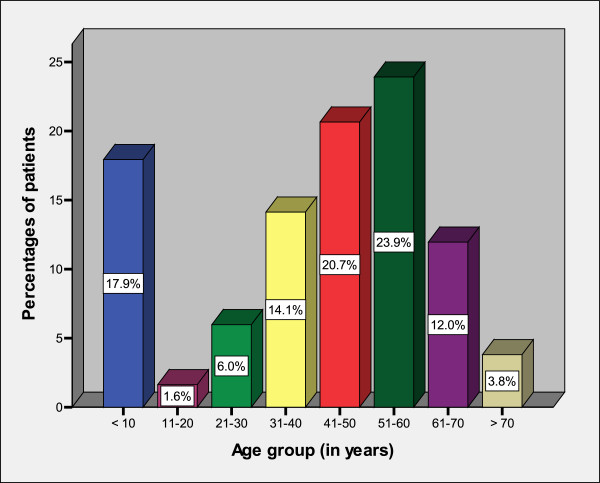
Distribution of patients according to age group.

During the study period, a total of 184 patients of gastric outlet obstruction were enrolled. The age of patients at presentation ranged from 2 weeks to 78 years with a median age of 46 years. The modal age group in children was 0 – 10 years (median 2 weeks) and in adult it was 51–60 (median 52 years). Patients aged ten years and below accounted for 33 (17.3%) patients (Figure 1). Of these, 26 (78.8%) patients were aged below two months. The median age of patients with benign causes was 34 years (range 2 weeks to 46 years), while that of malignant causes was 56 years (range 42 to 78 years). The difference in age distribution of the benign and malignant disease was statistically significant (P < 0.001). There were 122 (66.3%) males and 62 (33.7%) were females with a male to female ratio of 2: 1. Both the benign and malignant gastric outlet obstruction was found to be more commonly amongst the males than females. The male to female ratio for benign gastric outlet obstruction was 1.2: 1, while it was 3.2: 1 for the malignant gastric outlet obstruction. This difference was statistically significant (P < 0.001). The majority of patients, 156 (84.8%) came from the rural areas located a considerable distance from the study area and more than 80% of them were unemployed. Most of our patients, 149 (81.0%) had either primary or no formal education and the vast majority of them, 175 (95.1%) had no identifiable health insurance.

### Etiological spectrum of gastric outlet obstruction

The etiology of gastric outlet obstruction was benign in 82 (44.6%) cases, whereas 102 (55.4%) patients had malignant cause. Peptic ulcer disease was the commonest cause among the benign group in 28.2% of patients, whereas the commonest cause among the malignant group was gastric cancer in 42.9% of patients. In children, the commonest cause of gastric outlet obstruction was pyloric stenosis accounting for 13.0% of cases (Table 1).

**Table 1:** Distribution of patients according to causes of gastric outlet obstruction

**Causes of gastric outlet obstruction**	**Number of patients**	**Percentage**
**Benign causes**	**82**	**44.6**
• Peptic ulcers	52	28.3
• Hypertrophic pyloric stenosis	24	13.0
• Gastric polyp	2	1.1
• Caustic ingestion	2	1.1
• Latrogenic	1	0.5
• Gastric/duodenal tuberculosis	1	0.5
• Prepyloric web	1	0.5
**Malignant causes**	**102**	**55.4**
• Gastric cancer	79	42.9
• Carcinoma pancreas	16	8.7
• Periampullary Ca	3	1.6
• Cholangiocarcinoma	3	1.6
• Duodenal carcinoma	1	0.5

### Clinical presentation of patients with gastric outlet obstruction

The duration of illness ranged from 1 week to 8 years with a median duration of 6 months respectively. The time interval between symptom onset and diagnosis was often more than 6 months in the majority of patients (148, 80.4%). The clinical presentation of gastric outlet obstruction is shown in Table 2. Previous history of peptic ulcer disease was reported in 35 (19.0%) patients. Patients with a previous history of peptic ulcer disease had had symptoms for durations ranging from six months to 10 years (median 2 years) and all of them were not on regular anti-ulcer therapy. History of alcohol and smoking was reported in 104 (56.5%) and 67 (36.4%) patients respectively.

**Table 2:** Distribution of patients according to clinical presentation

**Clinical presentation**	**Frequency**	**Percentage**
Non-bilious vomiting	184	100
Weight loss	172	93.5
Succession splash	144	78.3
Bloating/epigastric fullness,	104	56.5
Epigastric pain	104	56.5
Dehydration	101	54.9
Epigastric mass	46	25.0
Shock	23	12.5

In this study, nine (4.9%) patients had associated co-morbid illness namely tuberculosis in 3 patients and hypertension, diabetes mellitus and sickle cell disease in 2 patients each respectively. Eighteen (9.8%) patients were HIV positive. Of these, 5 (27.8%) patients were known cases on ant-retroviral therapy (ARV) and the remaining 13 (72.2%) patients were newly diagnosed patients.

### Investigations among patients with gastric outlet obstruction

One hundred thirty-two (71.7%) of the patients had plain abdominal x-ray films available for review and demonstrated gastric air-fluid levels in 102 (77.3%) patients. Barium meal and follow through performed in 47 (25.5%) revealed an enlarged stomach and pyloroduodenal stenosis in 42 (89.4%) patients. Upper gastrointestinal endoscopy (oesophagogastroduodenoscopy) performed in 154(83.7%) revealed positive results in all patients (100%) and this was diagnostic. Abdominal ultrasound and Computerized tomography (CT) scan performed in 89 (48.4%) and 18 (9.8%) patients demonstrated positive results in 82 (92.1%) and 18 (100%) patients respectively. Complete Blood Count, Hemoglobin levels and ESR were done in all patients. More than eighty percent of the patients had Hemoglobin levels less than 10.0 gm/dl and ESR in the first hour was found ranging between 12–148 mm. Serum electrolytes performed in all patients revealed *hypokalemic hypochloremic metabolic alkalosis* in 106 (57.6%) patients. Serum albumin done in 126 (68.5%) patients revealed hypoalbuminaemia in 108 (58.7%) patients. HIV status was known in all patients and revealed positive results in 18 (9.8%) patients. CD 4+ count among HIV positive patients was available in only 15 patients and ranged from 102 cells/μl to 745 cells/μl with the median CD 4+ count of 282 cells/μl. A total of eight (44.4%) HIV positive patients had CD4+ count below 200 cells/μl and the remaining 10 (55.6%) patients had CD4+ count of ≥200 cells/μl.

### Pre-operative anaesthetic assessment and admission patterns

**Figure 2:**
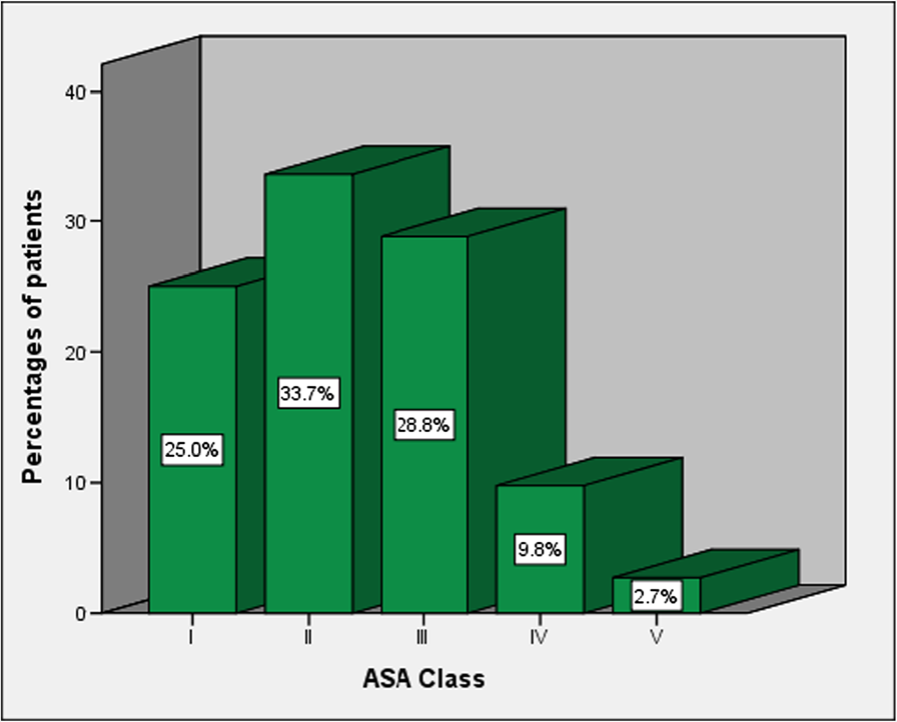
Distribution of patients according to ASA classification.

All patients who were scheduled for operation (168) were assessed pre-operatively using the American Society of Anesthetists (ASA) pre-operative grading (Table 3). The majority of patients had ASA class II accounting for 33.7% of cases (Figure 2). A high ASA class was found to be an independent predictor of mortality (p = 0.003).

**Table 3:** American Society of Anesthetists (ASA) classification

**ASA class**	**Description**
I	Healthy individual with no systemic disease
II	Mild systemic disease not limiting activity
III	Severe systemic disease that limits activity but is not incapacitating
IV	Incapacitating systemic disease which is constantly life threatening
V	Moribund, not expected to survive 24 hours with or without operation

Note: E is added to the class when the case is an emergency e.g. IIE refers to ASA class scheduled for emergency surgery.

The majority of patients, 142 (77.2%) were admitted through surgical outpatient clinic and the remaining 42 (22.8%) patients were admitted through the Accident & Emergency department. Thirty-eight (20.7%) patients were, after operation, admitted in the Intensive Care Unit (ICU) before being admitted to the general surgical wards. Out of these, 30 (78.9%) were subjected to ventilatory support for a median duration of 8 days (range 1–14 days).

### Treatment modalities

A total of 168 (91.3%) patients underwent surgery. Of these, gastro-jejunostomy was the most common surgical procedure performed accounting for 61.9% of cases (Table 4). Fourteen (7.6%) patients were treated successively with Histamine-2 (H2) blockers and proton pump inhibitors. The remaining two (1.2%) patients were unfit for surgery due to advanced disease. One patient, who had gastro-duodenal tuberculosis, after histological conformation of tuberculosis, was treated postoperatively with anti-tuberculous drugs with good results.

**Table 4:** Distribution of patients according to type of surgical procedure (N= 168)

**Type of surgical procedure**	**Frequency**	**Percentage**
Gastro-jejunostomy	104	61.9
Ramstedt’s operation (pyloromyotomy)	24	14.3
Gastrectomy	22	13.1
Laparotomy ± biopsy only (for malignant obstruction)	7	4.2
Truncal vagotomy	5	3.0
Heineke-Mikulicz pyloroplasty	4	2.4
Gastric polyp excision	2	1.2

### Treatment outcome

Fifty-four (32.1%) patients had 68 post-complications as shown in Table 5. Surgical site infection was the most common post-operative complication accounting for 38.2% of cases.

**Table 5:** Distribution of patients according to post-operative complications (N= 68)

**Post –operative complications**	**Frequency**	**Percentage**
Surgical site infection	26	38.2
Postoperative pyrexia	10	14.7
Pneumonia	8	11.8
Postoperative vomiting	6	8.8
Afferent loop syndrome	4	5.9
Dumping	4	5.9
Diarrhea	3	4.4
Deep venous thrombosis	3	4.4
Urinary tract infection	2	2.9
Paralytic ileus	1	1.5
Peritonitis	1	1.5
**Total**	**68**	**100**

The overall length of hospital stay (LOS) ranged from 4 to 72 days with a median of 14 days. The median LOS for non-survivors was 7 days (range 1-14 days). Patients who developed post-operative complications stayed longer in the hospital and this was statistically significant (P = 0.002).

In this study, thirty-four patients died giving a mortality rate of 18.5%. According to multivariate logistic regression analysis, age > 60 years (OR = 2.3, 95% C.I. (1.2-6.9), p= 0.003), co-existing medical illness (OR = 8.5, 95% C.I. (2.5-18.9), p = 0.011), malignant cause (OR = 1.3, 95% CI (1.9- 8.4), p = 0.021), HIV positivity (OR = 2.9, 95% CI (1.1- 8.8), p = 0.012), low CD 4 count (<200 cells/μl) (OR = 2.0, 95% CI (1.9-10.5), p = 0.001), high ASA class (OR = 8.1, 95% CI (2.6-12.7), p = 0.014), surgical site infection (OR = 4.5, 95% CI (1.1-8.6), p = 0.022) were the main predictors of mortality.

### Follow up of patients

**Figure 3:**
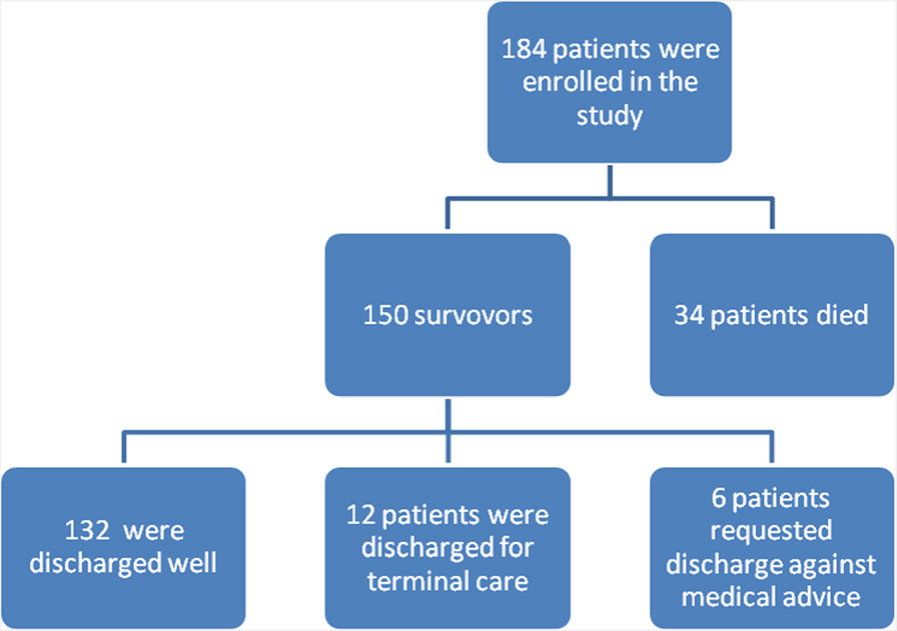
Distribution of patients according to follow up of patients.

Out of 150 survivors, one hundred thirty-two (88.0%) patients were discharged well, twelve (8.0%) were discharged for terminal care and the remaining six (4.0%) patients were discharged against medical advice. No patient among survivors in this study had permanent disabilities (Figure 3). Of the 150 survivors, fifty-four (36.0%) patients were available for follow up at three to six months after discharge and the remaining 96 (64.0%) patients were lost to follow up.

## Discussion

Gastric outlet obstruction poses diagnostic and therapeutic challenges to general surgeons practicing in resource-limited countries and contributes significantly to high morbidity and mortality [1-6]. This study was conducted in our environment to describe our own experiences in the management of this challenging disease; the problem not previously studied at our centre or any other hospital in the country. In this review, the highest age incidence of the patients at presentation was in the fourth decade of age and males were more affected. Most of the patients with benign gastric outlet obstruction in our study were in younger age group while malignant causes were in elder age group. The incidence of malignant gastric outlet obstruction in patients of older age group was also reported by others [1,7,13]. Our demographic profile is in sharp contrast to what is reported in other studies [6,9] where the majority of the patients are in the fifth and sixth decade of life. This discrepancy in age incidence may be attributed to by the large number of children in the current study. We could not establish the reasons for the male predominance.

Gastric outlet obstruction has been reported to be more prevalent in people with low socio-economic status [6]. This is reflected in our study where most of patients had either primary or no formal education and more than seventy-five percent of them were unemployed. The majority of patients in the present study came from the rural areas located a considerable distance from the study area and more than eighty percent of them had no identifiable health insurance. Similar observation was reported by others [6,13]. This observation has an implication on accessibility to health care facilities and awareness of the disease.

The majority of patients in this study had malignant gastric outlet obstruction which is in agreement with other studies reported elsewhere [1,3-5], but at variant with Kolisso [6] in Ethiopia who reported benign gastric outlet obstruction (peptic ulcer disease) as the most common cause of gastric outlet obstruction. In our study, gastric cancer was the commonest cause of malignant gastric outlet obstruction while peptic ulcer disease was the commonest benign cause. This is keeping with other studies which reported similar etiological pattern [1,13,14]. The predominant causes of gastric outlet obstruction have changed substantively with the identification of *Helicobacter pylori* and the use of proton pump inhibitors. Until the late 1970s, benign disease was responsible for the majority of cases of gastric outlet obstruction in adults, while malignancy accounted for only 10 to 39 percent of cases [2,13]. By contrast, in recent decades, 50 to 80 percent cases have been attributable to malignancy [2,13,15,16].

The clinical presentation of gastric outlet obstruction in our patients is not different from those in other studies [2-7], with non-bilious vomiting being common to all the patients. In keeping with other studies [6,16], the majority of our patients had symptoms of more than 6 months duration at the time of presentation. Late presentation in our study may be attributed to lack of accessibility to health care facilities and lack of awareness of the disease. It is worth noting that seventeen (18%) of our gastric outlet obstruction patients secondary to complicated peptic ulcer had no previous history of ulcer symptoms prior to the onset of illness. Patients with no previous diagnosis of peptic ulcer have a higher risk of developing complications such as gastric outlet obstruction than patients with a known history of ulcer disease. This may be because preventative measures are more likely to have been taken in patients with a known history of peptic ulcer disease. Furthermore, these patients are perhaps more likely to seek treatment earlier.

The presence of co-existing medical illness has been reported elsewhere to have an effect on the outcome of patients with gastric outlet obstruction [6]. This is reflected in our study where patients with co-existing medical illness had significantly high mortality rate.

The prevalence of HIV infection in the present study was 9.8%, a figure that is significantly higher than that in the general population in Tanzania (6.5%) [17]. This difference was statistically significant (P < 0.001). However, failure to detect HIV infection during window period and exclusion of some patients from the study may have underestimated the prevalence of HIV infection among these patients. We could not establish the reason for the high HIV seroprevalence in our study population and we could not find any literature regarding the effect of HIV infection on the etiology and outcome of patient with gastric outlet obstruction. This calls for a need to research on this observation. In this study, HIV infection was found to be associated with poor postoperative outcome. This observation calls for routine HIV screening in patients with gastric outlet obstruction.

In agreement with other studies [1,6], the diagnosis of gastric outlet obstruction in this study was based on clinical presentation, an upper gastrointestinal barium study, and/or an inability during upper endoscopy to intubate the second portion of the duodenum (upper gastrointestinal endoscopy) and confirmed by histology and intra-operative findings. Other diagnostic investigations included abdominal ultrasound and computerized tomography (CT) scan.

The treatment of gastric outlet obstruction depends on the cause, but is usually either surgical or medical. In most patients with peptic ulcer disease, the edema will usually settle with conservative management with nasogastric suction, replacement of fluids and electrolytes and proton pump inhibitors [8]. Surgery is indicated in cases of gastric outlet obstruction in which there is significant obstruction and in cases where medical therapy has failed [8,18]. In the current study, gastro-jejunostomy was the most frequent type of surgical procedure performed. This is in line with other studies done elsewhere [19-21]. The high rate of gastro-jejunostomy in our study is attributed to the large number of patients with malignant gastric outlet obstruction. Traditionally, malignant gastric outlet obstruction has been treated surgically, usually by creating a gastro-jejunostomy. More recently, the use of endoscopically placed self-expandable metal stents (SEMS) has become a routine practice [9,10]. However, this procedure was not popular in our study due to lack of this facility in our centre.

The presence of complications has an impact on the final outcome of patients presenting with gastric outlet obstruction [18]. In our review, the postoperative complication rate was 32.1%, a figure which is higher than that reported by other authors [22,23]. In agreement with other studies [6,22,23], surgical site infection was the most common postoperative complications in the present study. High rate of surgical site infection in this study may be attributed to HIV seropositivity and low CD 4 count.

The overall median duration of hospital stay in the present study was 14 days which is higher than that reported by Kolisso [6] in Ethiopia. This can be explained by the presence of large number of patients with postoperative complications in our study. However, due to the poor socio-economic conditions in Tanzania, the duration of inpatient stay for our patients may be longer than expected. Prolonged duration of hospital stay has an impact on hospital resources as well as on increased cost of health care, loss of productivity and reduced quality of life.

The overall mortality rate in this study was 18.5% and it was significantly associated with the age > 60 years, co-existing medical illness, malignant cause, HIV positivity, low CD 4 count (<200 cells/μl), high ASA class and presence of surgical site infection. Addressing these factors responsible for high mortality in our patients is mandatory to be able to reduce mortality associated with this disease.

Self discharge by patient against medical advice is a recognized problem in our setting. Similarly, poor follow up visits after discharge from hospitals remain a cause for concern. These issues are often the results of poverty, long distance from the hospitals and ignorance and need to be addressed.

Delayed presentation and the large number of loss to follow up were the major limitations in this study. However, despite these limitations, the study has provided local data that can be utilized by health care providers to plan for preventive strategies as well as establishment of management guidelines for these patients. The challenges identified in the management of patients with gastric outlet obstruction in our environment need to be addressed, in order to deliver optimal care for these patients.

## Conclusion

Gastric outlet obstruction is a common surgical problem in our setting and poses diagnostic and therapeutic challenges. It is more common among males with malignant causes being more prevalent. The benign gastric outlet obstruction is seen in young patients while malignant causes in elder age group. Gastric cancer is the commonest malignant cause of gastric outlet obstruction whereas peptic ulcer diseases the commonest benign etiology. In children, congenital pyloric stenosis is the commonest cause of gastric outlet obstruction. The majority of patients present late with poor general condition. Gastro-jejunostomy is the most common surgical procedure performed. The result of this study suggests that early recognition of the diagnosis, aggressive resuscitation and early institution of surgical management is of paramount importance if morbidity and mortality associated with gastric outlet obstruction are to be avoided.

## Competing interests

The authors declare that they have no competing interests.

## Authors’ contributions

HJ and PLC conceived the study and participated in the literature search, writing of the manuscript and editing the article. PLC submitted the manuscript and is the corresponding author. MDM and PFR participated in study design, data analysis, manuscript writing & editing. In addition, HJ did the endoscopic examination and PFR did the histopathological examination. All the authors read and approved the final manuscript.

## Pre-publication history

The pre-publication history for this paper can be accessed here:

http://www.biomedcentral.com/1471-2482/13/41/prepub
